# Dose-Associated Changes in Gait Parameters in Response to Exercise Programs after Total Knee Arthroplasty: Secondary Analysis of Two Randomized Studies

**DOI:** 10.4172/2329-9096.1000311

**Published:** 2015

**Authors:** Sara R Piva, Shawn Farrokhi, Gustavo Almeida, Kelley Fitzgerald G, Timothy J Levison, Anthony M DiGioia

**Affiliations:** 1; 2

**Keywords:** Gait, Exercise, Rehabilitation, Gait parameters, Osteoarthritis

## Abstract

**Background:**

Rehabilitation plays an important role to improve the outcomes of total knee arthroplasty (TKA). Evidence about the appropriate dose of exercise to recover gait dysfunction after TKA is limited. We posed the research question: In patients during the post-acute stage after TKA, is increased dose of exercise associated with larger improvements in gait parameters such as step length and single support time?

**Methods:**

This was a secondary analysis from two randomized studies on exercise after TKA to investigate dose-dependence of gait parameters in response to exercise. Participants were 50 years or older who underwent unilateral TKA at least two months prior. They participated in 2 months of supervised exercises followed by 4 months of a home exercise program. The primary outcome was change in gait parameters from baseline to 6 months. Participants were divided in three groups according to the dose of exercise: group 1 (light-to-moderate intensity exercise), group 2 (high intensity + functional exercise), and group 3 (high intensity + functional + balance exercise). Jonckheere-Terpstra test was used to test if the magnitude of changes in gait parameters increased from group 1 to group 3 in an ordered fashion.

**Results:**

Increased dose of exercise was associated with progressive increases in step length in the operated-limb (p=0.008) and decreases in step length in the non-operated limb (p=0.011). Increased dose of exercise was associated with ordinal decreases in loading response time (p=0.049) and increases in single-leg support time (p=0.021) on the operated- limb, but not on the non-operated-limb. Increased dose of exercise was associated with decreases in unloading time on the non-operated-limb (p=0.011) but not on the operated-limb (p=0.400).

**Conclusions:**

Significant dose-response of exercise on gait parameters support the promotion of more intensive exercise programs that combine functional and balance training programs after TKA.

## Introduction

Knee osteoarthritis (OA) is among the most disabling medical conditions in the world’s ageing population [1-4]. However, there is no clear consensus on how knee OA should be effectively managed [5-9]. Given the lack of long-term treatment options, knee OA leads to total knee arthroplasty (TKA) as the last option to relieve pain and improve function. Estimates suggest that around 1.3 million TKA surgeries are performed annually around the world [10].

While TKA has shown to be a cost-effective treatment for alleviating pain and restoring physical function [11], some gait alterations such as decreased support time and shorter step length in the operated limb, are known to persist for months or years after the TKA surgery [12-20]. From a biomechanical point of view, these persistent gait alterations are important as they may lead to a shift in relative load bearing from the operated limb on to the non-operated limb, which may increase the rate of disease progression reported after TKA [16,20]. From a clinical standpoint, normal gait is crucial for daily activities and one of the main goals of patients and health care providers after TKA.

Rehabilitation plays an important role in functional recovery after TKA. However, effective rehabilitation approaches to improve gait alterations after TKA remain elusive. It has been suggested that high-intensity resistance training along with functional and balance exercises are effective to improve functioning after TKA [21-26]. Empirical observation of these studies indicates a dose-dependence between exercise and outcome. Yet, there is currently no evidence to help clinicians select the appropriate dose of exercise to improve gait after TKA. To that end, we conducted two randomized studies on exercise after TKA that tested different strategies of exercise that could be combined to investigate this dose-dependence. One study tested the effectiveness of a high-intensity exercise program that combines functional and balance exercises compared to usual care exercises on physical function [27]. The other study investigated whether a functional exercise program supplemented with balance exercises improve physical function compared to functional exercise only [22]. This article reports the results of post hoc analyses of these studies to answer the following question: In patients who are in the post-acute stage after TKA, is increased dose of exercise associated with larger improvements in gait parameters such as step length and single support time of the operated and non-operated limbs? We hypothesized that significant dose-associated changes would be identified for spatial and temporal gait parameters.

## Materials and Methods

A complete description of the studies included in this analysis is detailed in the primary publications [22,27]. Only the methodological details related to the post hoc analysis are provided here. The two studies were prospective randomized clinical studies implemented in the rehabilitation clinic of the Department of Physical Therapy at the University of Pittsburgh. All subjects signed consent forms approved by the University of Pittsburgh Institutional Review Board. Sample size was not estimated for this study as it reports on post hoc analyses.

Subjects from both studies were recruited through letters sent to patients who had undergone a tri-compartmental cemented TKA performed by the same surgeon. After surgery, all participants received the same rehabilitation while in the hospital and outpatient physical therapy as needed prior to study participation. Participants who were interested in the study called the study coordinator who fully explained the study, obtained consent, and examined subjects for eligibility.

Inclusion criteria were age above 50 years and a unilateral TKA done 2 to 6 months prior to starting the study. Subjects were excluded if they reported 2 or more falls within the previous year, were unable to ambulate a distance of 30 meters without an assistive device, had an acute illness, and had a history of cardiovascular disease, uncontrolled hypertension, severe visual impairment, lower-extremity amputation, or progressive neurological disorder. For this study, subjects were also required to have participated in the assessment of gait parameters.

Subjects were tested at baseline and 6 months after randomization by testers who were masked to study assignment. During baseline the subjects completed demographics, pain, and physical function questionnaires, which were used to describe the sample. Pain intensity in the surgical knee was assessed using an 11-point numeric pain scale anchored on the left with the phrase “No pain” and on the right with the phrase “Worst imaginable pain” [28,29]. Physical function was assessed by Western Ontario and McMaster Universities (WOMAC) Osteoarthritis Index [30-32].

Gait parameters were the main outcome of this study and were tested at baseline and 6 months. Gait parameters were measured using a reliable and valid electronic walkway (GaitRite^®^ v 3.8, CIR Systems Inc., New Jersey, USA) connected to a portable computer [33-36]. Subjects were asked to walk at a self-selected speed during 8 successive passes across the walkway while wearing shoes. They started and finished walking 1.5 meters in front of and beyond the edge of the walkway to avoid acceleration/deceleration. Data collected from the 8 trials were averaged. Gait parameters included step length and three temporal parameters corresponding to the three phases of stance: loading response, single-leg support, and unloading times. Step length was measured in centimeters whereas temporal parameters were measured in seconds. Since gait parameters are affected by gait velocity, the gait data were adjusted by gait velocity in order to account for potential changes in gait velocity from pre to post intervention. Thus, step length is reported as a percentage of the stride length (step length/stride length) and temporal parameters are reported in percentage of the gait cycle (e.g., loading response/gait cycle). Because this study investigates dose-response to exercise, the variable of interest is the change in gait parameters (6-month value minus baseline value).

**Figure 1:**
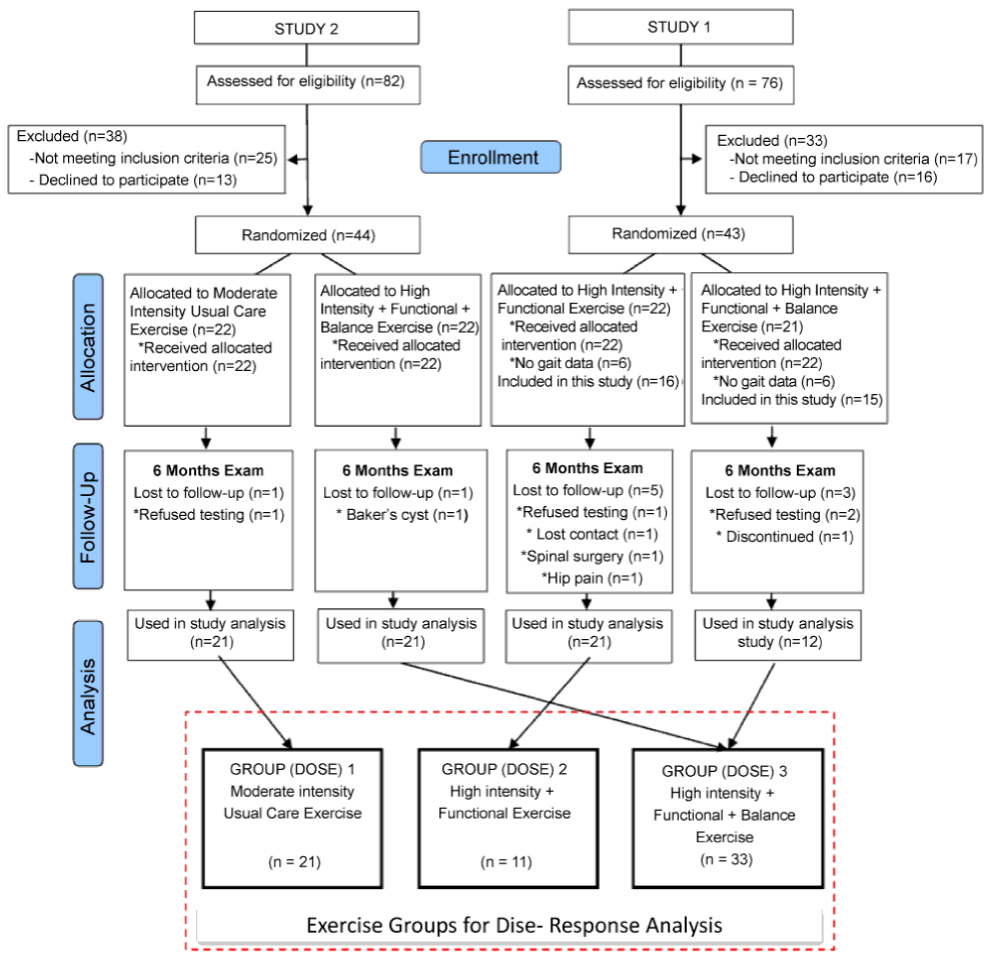
Flow Diagram of Two Studies used for this Post-hoc Analysis.

Randomization was done after baseline assessment by a research assistant not involved with recruitment and was stratified by gender and age in random block sizes of two and four. A statistician not involved with study enrollment generated the randomization plan using a validated computer program. In both studies, subjects were randomly assigned to one of two treatment groups. In study 1, the groups were a) Light-to-moderate Intensity Exercise or b) High-Intensity + Functional + Balance Exercise. In study two, the groups were c) High-Intensity + Functional Exercise or d) High-Intensity + Functional + Balance Exercise. To test our hypothesis, groups ‘b’ and ‘d’ were combined as one group since the exercise programs were then defined in an orderly fashion according to increased dose of exercise. Group ‘a’ (light-to- moderate intensity exercise) was defined as Group 1, group ‘c’ (high-intensity + functional exercise) was defined as Group 2, and groups ‘b’ and ‘d’ were combined and defined as Group 3 (high-intensity + functional + balance exercise)(Figure 1). All participants completed 12 exercise sessions supervised by physical therapists spread over 2 months, followed by 4 months of a home exercise program, totaling 6 months of exercise. The exercises were done on both legs.

### Group 1: light-to-moderate intensity exercise

This exercise program included warm-up, light-to-moderate intensity aerobic exercise, and resistance exercise. Warm-up consisted of 15 minutes of lower extremity stretching, range of motion, and stationary bike without resistance (Table 1). Aerobic training consisted of 20 minutes of light-intensity treadmill walking between 40-50% of age estimated maximal heart rate. Resistance exercise targeted knee extensors, knee flexors, hip extensors, and hip abductors (Table 1). Exercises used weight machines during supervised visits, whereas ankle weights and elastic bands were used for the home exercise program. The exercises using the weight machines were performed from light to moderate intensity, between 40-50% of 1 repetition maximum (1 RM). The level of effort using elastic bands and ankle weights as resistance was appraised by a perceived exertion scale with rates from light to moderate [37,38]. Subjects performed 2 sets of 20 repetitions of each exercise without reaching fatigue. Resistance exercise took around 40 minutes, totalling approximately 75 minutes of exercise for each session in this group.

### Group 2: high-intensity plus functional exercise

Subjects performed the same warm-up as described for Group 1, followed by high-intensity aerobic and resistance exercise, and functional task-oriented training. Aerobic training consisted of 20 minutes of treadmill walking between 50-75% of the age estimated maximal heart rate. The resistance exercise program was the same as in Group 1 but at a higher intensity. For weight machines, all exercises were performed at high intensity; between 60 - 80% of 1 RM. The level of effort using elastic bands and ankle weights as resistance was appraised by a perceived exertion scale with rates from moderate to vigorous. Subjects performed 2 sets of 8 repetitions of each exercise reaching fatigue [37,38]. Resistance exercises took around 20 minutes. Functional task-oriented exercises consisted of 10 minutes of bilateral and unilateral mini squats, chair rises, and stair climbing (Table 1). The exercise session took approximately 65 minutes.

### Group 3: high-intensity plus functional exercise plus balance exercise

Subjects received the same program as described in Group 2, plus balance exercises that consisted of side stepping, tandem walking, cross-over steps during forward and backward walking, forward and backward walking to designated markers, multiple changes in direction, and standing over unstable surfaces (foam, tilt boards) (Table 1). These exercises took approximately 10 minutes, totaling around 75 minutes of exercise in this group.

## Data Analysis

Differences in baseline characteristics across the groups were determined by Analysis of Variance (ANOVA) or Kruskal-Wallis, for continuous variables with normal or non-normal distribution respectively. Data normality was assessed by Shapiro-Wilk test. Chi square test was used for nominal variables. We also visually inspected these data for differences that could be clinically important but did not reach significance.

To test the hypothesis of significant dose-associated changes for spatial and temporal gait parameters, we first calculated the mean change for each gait parameter and then utilized the non-parametric test for ordered alternatives (Jonckheere-Terpstra for k samples). Johckheere-Terpstra test assesses the alternative that the magnitude of change in gait parameters increases from group 1 to group 3 in an ordered fashion. Jonckheere-Terpstra is the most appropriate and powerful test to use when samples are expected to have a natural ordering (i.e., the parameter of the first group is smaller than the second, which in turn is smaller than (i.e., the parameter of the first group is smaller than the second, which in turn is smaller than for ordinal trends of the 3 different dose groups. Statistical significance was defined as 2-tailed p<0.05. Analyses were performed using IBM SPSS v22.0 software (Armonk, NY).

## Results

Details of recruitment and retention are available in Figure 1. Study one took place from Jan/2007 to May/2008 and study two from Oct/ 2011 to Aug/2013. Both studies stopped when target recruitment was reached. Of the 43 subjects enrolled in study one, 12 were excluded from analysis due to the lack of gait data because the gait mat was purchased after these first 12 subjects had been recruited. Additionally, 8 subjects in study one were not available during the 6-month follow-up and gait parameters could not be obtained. Of the 44 subjects enrolled in study two, two were not available during the 6-month follow-up. Subjects in the three intervention arms attended similar number of supervised exercise sessions, with an average of 11.5 out of 12 sessions (96%). There were no adverse events in both studies.

Both visual inspection and ANOVA results revealed no differences between the groups for demographic and biomedical characteristics (Table 2). Subjects in the three groups had low to moderate levels of pain and functional limitation.

**Figure 2:**
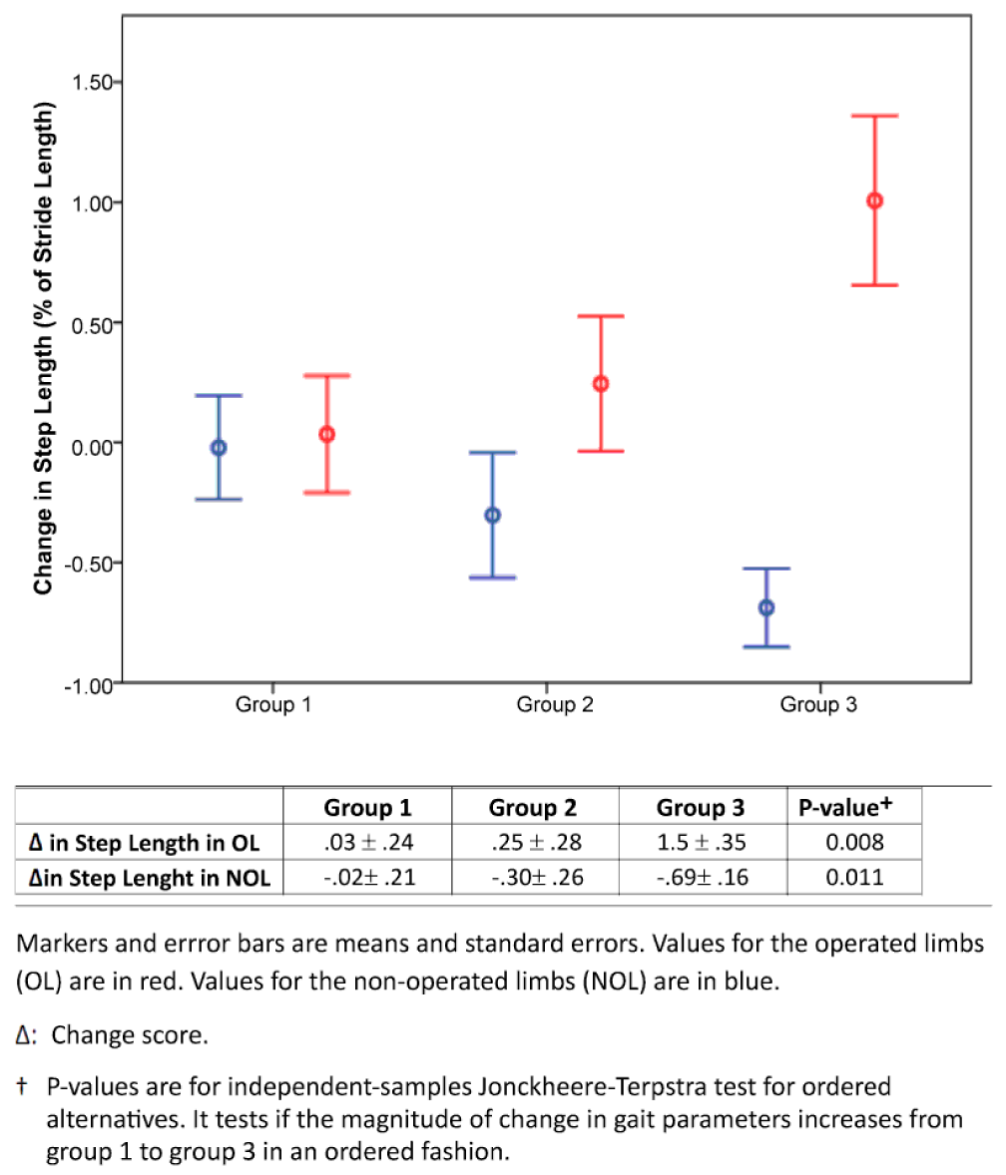
Changes in Step Length at 6 Months in the Three Exercise Dose Groups.

Figure 2 represents the changes in step length for the three exercise groups along with results from Jonckheere-Terpstra tests. Significant dose-associated changes were identified for step length in the operated and non-operated limbs, but in opposite directions. Increased dose of exercise was associated with progressively larger increases in step length in the operated limb, whereas increased dose of exercise in the non-operated limb led to progressive larger decreases in step length.

**Figure 3:**
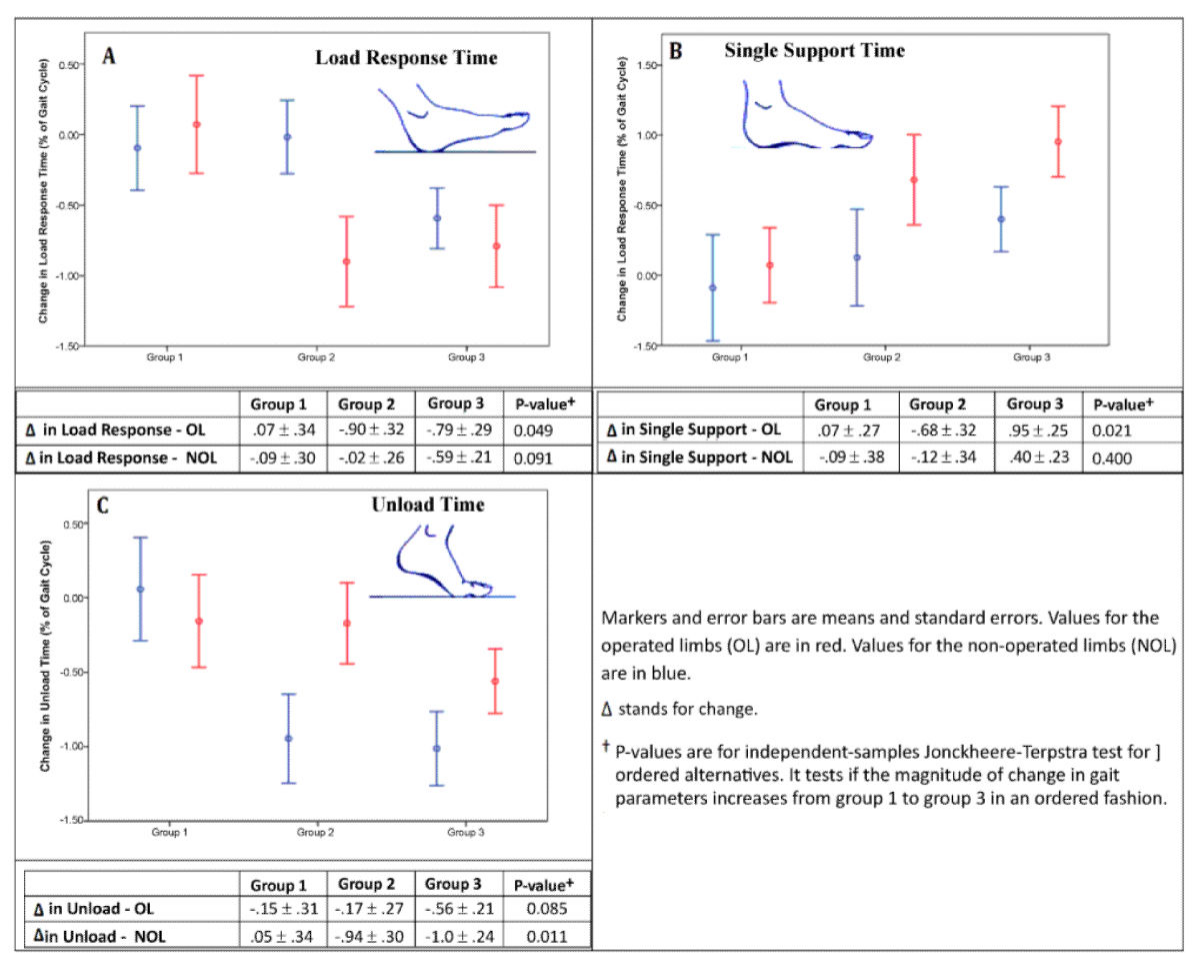
Changes in Load Response (Panel A), Single Support (Panel B) and Unload Times (Panel C) in the Three Exercise Dose Groups at 6 Months.

Figure 3 provides data for changes in loading response (Panel A), single-leg support (Panel B), and unloading (Panel C) times for the three exercise groups, along with results from Jonckheere-Terpstra tests. Significant dose-associated changes were identified for loading response, single-leg support, and unloading times. Increased dose of exercise was significantly associated with ordinal decreases in change in loading response time in the operated limb but not in the non-operated limb. Increased dose of exercise was also significantly associated with a progressive increase in change in single-leg support time in the operated limb. Although changes in single-leg support time in the non-operated limb was somewhat larger in group 3 as compared to group 2, which in turn was larger than group 1, the trend was not significant. Last, increased dose of exercise was significantly associated with progressive decreases in unloading time, but this trend was only significant in the non-operated limb.

## Discussion

To the best of our knowledge, this is the first study to investigate the dose-response of exercise programs on improvements in gait parameters in individuals after TKA. Our hypotheses of significant dose-associated changes for spatial and temporal gait parameters were affirmed. More specifically, ordinal increases in dose of exercise was associated with progressive increases in step length in the operated limb, and decreases in the non-operated limb; decreases in loading response time and increases in single-leg support time in the operated limb; and decreases in unloading time in the non-operated limb. Since gait alterations after TKA have been characterized by decreased support time and shorter step length in the operated limb, the findings of increased step length and single support time in the operated limb are positive ones and indicate beneficial effects of high exercise dose [12,14]. Thus, the findings provide credence to previous reports indicating that more intensive rehabilitation programs lead to clinically meaningful improvements in outcome during the later stages of recovery after TKA [21,22,24-26].

The longitudinal studies of pre and post TKA indicate that pre-surgery gait abnormalities are usually retained up to 18 months after surgery despite improvements in pain and range of motion [40]. As such, development or retention of gait alterations after TKA have been linked to the predictable patterns of further deterioration of the contralateral non-operated knee and the other lower limb joints [15,16]. If not treated appropriately, gait alterations observed after TKA, particularly those associated with joint loading, may contribute to the biomechanical factors that increase the rate of degeneration in the non-operated limb [16,41]. Even minor alteration in weight-bearing of the non-operated limb could have significant effects on disease status, as the average patient’s walking activity after TKA approaches approximately 1-2 million gait cycles per year [42,43]. As such, considering the small alterations of the different components of the gait cycle may present a useful composite picture of repetitive or prolonged loading of the non-operated limb. The achievement of progressive faster load response and longer single support time in the operated limb with higher doses of exercise may be a prerequisite to decrease joint loading on the non-operated limb and prevent later disease or joint injury on the non-operated limb. To date, longitudinal studies have not examined the potential deleterious effects of gait alterations on the non-operated limb. Future longitudinal studies in the area of walking biomechanics and rehabilitation should pay particular attention to the non-operated limb after TKA.

Despite the widespread use of TKA, there is a notable lack of consensus regarding which rehabilitative post-operative practices should be employed in this patient population [10,24]. Given the significant clinical deficits in activities of daily living such as gait and stair climbing reported more than one year after TKA [44,45], intensive rehabilitation programs that include supervised training sessions have been recommended in the sub-acute recovery period after TKA to optimize functional ability and quality of life [21,22,24,26]. The feasibility of a more intensive rehabilitation program has been further substantiated with reports of high exercise adherence and satisfaction with low dropout rate and adverse events [21,22,27]. Therefore, the above evidence combined with the findings of the current study justifies the use of intense muscular strengthening, aerobic training, and functional training combined with balance exercises after TKA.

This study has limitations. Although this study supports a dose-response of exercise programs on improvements in gait parameters, the impact of these changes on knee joint kinematics or kinetics were not evaluated. Future work should consider evaluating the dose-response effects of exercise on the changes in the overall biomechanical characteristics of both operated and non-operated limbs. Moreover, the changes in spatiotemporal gait parameters from pre to post intervention, although significantly dependent on the dose of exercise, seemed rather small. The changes in gait parameters reported in the current study could not be directly compared to those of previous investigations as the raw values were either not reported or normalized differently [12,14]. Furthermore, cut-offs of clinical meaningful changes in gait parameters have not yet been established, which makes it difficult to adjudicate clinical significance. Last, while the participants in the study represent the usual population of individuals during the sub-acute phase of TKA recovery, results of this study only apply to people who have undergone a first uncomplicated total knee surgery for severe OA [21,46]. It is not clear whether the same results could be obtained after bilateral TKA, revisions, or in individuals with other concurrent lower limb problems.

## Conclusion

The results of this study indicate that in patients in the post-acute stage after TKA, increased dose of exercise associate with larger improvements in gait parameters such as step length, loading response time, single-leg support time, and unloading time. The findings of significant dose-response of exercise combined with previous reports of better clinical outcomes after intense and comprehensive exercise programs justify promotion of more intensive exercise programs that combine functional training and balance programs after TKA. Further efforts are required to determine whether gait changes have an impact on the rate of further disease progression in the non-operated lower limb.

## Figures and Tables

**Table 1:** Description of Exercises.

**Warm-up**	Ankle range of motion-In long-sitting, ankle dorsal and plantarflexion.
	Knee range of motion-In long-sitting, flex the knee and hip as far as possible by sliding the foot toward the pelvis. Extension is done by sliding the foot back.
	Posterior leg stretching-In supine, with help of a belt, subject flexes exercise hip as far as possible keeping the knee in full extension (hold for 30 sec).
**Resistance training**	Knee extensors- Seated, long or short arc quadriceps. Exercise was performed from 90° to 60, from 30° to 0°, or terminal knee extension, depending on pain tolerance.
	Hamstrings curls in standing or lying prone (up to 60° of knee flexion).
	Hip abductors-side-lying with the back against a wall. Subject abducts the exercise hip ≈30°. The heel of the exercise limb touches the wall throughout the exercise. Ankle cuff weights are used for resistance.
**Functional tasks**	Get up and sit down from a chair. Initially use chair armrests for assistance and progress by not using armrest.
	Bilateral knee flexion/extension in standing. Start exercise bearing moderate body weight on handrail or counter, and progress not holding on handrail or counter. Exercise is also progressed into unilateral knee flexion/extension.
	Ascend and descend a flight of stairs. Progress speed as tolerated.
**Balance/ dynamic stability**	Side stepping. Progress by stepping over low obstacles.
	Braiding-alternate front and back cross-over steps while moving laterally (carioca).
	Tandem walk alternating legs with each step. Progress by stepping over low obstacles and by tandem walking backwards.
	Crossover walking-subject crosses one leg in front of the other, alternating legs with Each step to a maximum of ≈1ft width.
	Multiple change in direction-Therapist directs the subject to either walk forward, backward, sideways, or on diagonal by cueing patient with hand signals.
	Foam-subject stands on a soft foam surface with both feet on the ground. Therapist attempts to perturb patient balance in random fashion. Progress to single leg support.
	Tilt-board-subject stands on a tilt board with both feet on the board. The therapist perturbs the tilt board in forward and backward and side to side directions.

**Table 2:** Demographic and Biomedical Characteristics of Study Participants at Baseline.

	Group 1 n=21	Group 2 n=11	Group 3 N=33	p-value
Age - Mean ± SD	69 ± 6.0	70 ± 9.5	67 ± 6.6	0.402
Gender-N of Female (%)	12 (57)	8 (73)	27 (82)	0.142
Race-N (%)				0.353
Caucasian	20 (95)	11 (100)	29 (88)	
African-American	1 (5)	0 (0)	4 (12)	
Marital Status-N (%)				0.934
Married	12 (57)	7 (64)	20 (60)	
Not Married	9 (43)	4 (36)	13 (40)	
Education-N (%)				0.489
Less than College Degree	9 (43)	4 (36)	9 (27)	
College Degree	12 (57)	7 (64)	24 (73)	
BMI (kg/m2) - Mean ± SD	30 ± 4.4	31 ± 4.0	30 ± 4.2	0.775
Months Since Surgery- Median (Range)	3 (2)	2 (3)	2 (3)	0.451
Number of surgeries on right side (%)	11 (52)	5 (46)	17 (52)	0.926
Number of Comorbidities‡- Median (Range)	2 (5)	2 (5)	2 (6)	0.589
Knee Pain¥ - Mean ± SD	2.2 ± 1.4	2.1 ± 1.2	2.9 ± 2.6	0.300
Physical Function†- Mean ± SD	18.4 ± 10.9	20.7 ± 8.5	19.1 ± 9.1	0.814

‡ Number of Comorbidities was assessed as the number of health problems reported by the subjects including high blood pressure, stroke, diabetes, blood disorder, cancer, depression, back pain, memory problems, hip fracture, and lung, stomach, kidney, liver, or heart disease;

¥ Knee pain was assessed by an 11-point numeric pain scale;

† Physical function was assessed by the Western Ontario and McMaster (WOMAC) Universities Osteoarthritis Index;

The WOMAC physical function subscore has 17 items (each scored from 0 to 4) with possible scores from 0 to 68 points. Larger scores in WOMAC indicate worse pain and function. WOMAC version LK3.1 was used.
